# Identification of microRNA biomarker candidates in urine and plasma from rats with kidney or liver damage

**DOI:** 10.1002/jat.3358

**Published:** 2016-07-11

**Authors:** Francis S. Wolenski, Pooja Shah, Tomoya Sano, Tadahiro Shinozawa, Hugues Bernard, Matt J. Gallacher, Shylah D. Wyllie, Georgianna Varrone, Lisa A. Cicia, Mary E. Carsillo, Craig D. Fisher, Sean E. Ottinger, Erik Koenig, Patrick J. Kirby

**Affiliations:** ^1^ Drug Safety Research & EvaluationMillennium Pharmaceuticals, Inc., a wholly owned subsidiary of Takeda Pharmaceutical Company LimitedCambridgeMA02139USA; ^2^ Molecular PathologyMillennium Pharmaceuticals, Inc, a wholly owned subsidiary of Takeda Pharmaceutical Company LimitedCambridgeMA02139USA; ^3^ Drug Safety Research LaboratoriesTakeda Pharmaceutical Company LimitedFujisawaKanagawa251-8555Japan

**Keywords:** microRNA, miR‐seq, biomarker, hepatotoxicity, nephrotoxicity, bioinformatics

## Abstract

MicroRNAs (miRNA) are short single‐stranded RNA sequences that have a role in the post‐transcriptional regulation of genes. The identification of tissue specific or enriched miRNAs has great potential as novel safety biomarkers. One longstanding goal is to associate the increase of miRNA in biofluids (e.g., plasma and urine) with tissue‐specific damage. Next‐generation sequencing (miR‐seq) was used to analyze changes in miRNA profiles of tissue, plasma and urine samples of rats treated with either a nephrotoxicant (cisplatin) or one of two hepatotoxicants (acetaminophen [APAP] or carbon tetrachloride [CCL_4_]). Analyses with traditional serum chemistry and histopathology confirmed that toxicant‐induced organ damage was specific. In animals treated with cisplatin, levels of five miRNAs were significantly altered in the kidney, 14 in plasma and six in urine. In APAP‐treated animals, five miRNAs were altered in the liver, 74 in plasma and six in urine; for CCL_4_ the changes were five, 20 and 6, respectively. Cisplatin treatment caused an elevation of miR‐378a in the urine, confirming the findings of other similar studies. There were 17 in common miRNAs elevated in the plasma after treatment with either APAP or CCL_4_. Four of these (miR‐122, −802, −31a and −365) are known to be enriched in the livers of rats. Interestingly, the increase of serum miR‐802 in both hepatotoxicant treatments was comparable to that of the well‐known liver damage marker miR‐122. Taken together, comparative analysis of urine and plasma miRNAs demonstrated their utility as biomarkers of organ injury. Copyright © 2016 The Authors. *Journal of Applied Toxicology* published by John Wiley & Sons Ltd.

## Abstract 2

MicroRNAs (miRNA) have great potential as novel safety biomarkers. Next‐generation sequencing was used to analyze changes in miRNA profiles of tissue, plasma and urine samples of rats treated with either a nephrotoxicant (cisplatin) or one of two hepatotoxicants (acetaminophen or carbon tetrachloride). Cisplatin treatment caused an elevation of miR‐378a in the urine, confirming the findings of other similar studies. Treatment with either acetaminophen or carbon tetrachloride caused a serum elevation of four liver‐enriched miRNAs (miR‐122, −802, −31a and −365).

## Introduction

Biomarkers are critical for the pre‐clinical assessment of drug toxicity and provide information about which tissues are affected, the extent of damage, and provide a convenient way to monitor the injury as it increases in severity or recovers. Recent attention has focused on the use of microRNA (miRNA) as a novel class of predictive safety biomarkers (Ozer *et al.*, 2008). miRNAs are short 21–25 nucleotide RNAs that regulate the expression and function of much of the genome (He & Hannon, 2004). Certain miRNAs are highly enriched in one tissue (Etheridge *et al.*, 2011) and can be readily detected in biofluids after injury (Blondal *et al.*, 2013). In humans, urinary miRNA has been assessed in patients with kidney damage (Konta *et al.*, 2014; Maluf *et al.*, 2014). One example of an established predictive miRNA biomarker is miR‐122, which has been used to assess hepatotoxicity in rodents (Wang *et al.*, 2009), dogs (Harrill *et al.*, 2014; Koenig *et al.*, 2016) and humans (Starkey Lewis *et al.*, 2011).

The Sprague–Dawley strain of rat is an important non‐clinical species that is routinely used in toxicological evaluations of compounds. Whole body miRNA atlases rats were compiled using either a miRNA microarray platform (Minami *et al.*, 2014) or more recently a next‐generation sequencing (miR‐seq) approach (Smith *et al.*, 2016). Both technologies have their advantages/disadvantages that are extensively described elsewhere (Git *et al.*, 2010; Hurd & Nelson, 2009; Kelly *et al.*, 2013). However, one key limitation with arrays is that they are limited to preselected miRNAs, which can hamper the identification of novel miRNAs (Yang *et al.*, 2012). A direct comparison of both technologies in a rodent toxicity model concluded that miR‐seq had superior specificity and detected more miRNA isoforms than the array approach (Nassirpour *et al.*, 2014). Thus, miR‐seq appears to be the more robust platform for the detection of novel miRNA biomarkers.

The goal of this study was to dose rats with toxicants that caused nephrotoxicity or hepatotoxicity, and then identify changes within the miRNA expression profiles in urine, plasma and tissue. Kidney damage was caused by cisplatin, a chemotherapeutic, and liver damage was induced by either an over‐the‐counter pain reliever acetaminophen (APAP) or the industrial solvent carbon tetrachloride (CCL_4_). Single doses of these three classical toxicants were previously shown in rats to cause organ‐specific damage (Kanki *et al.*, 2014; Yang *et al*., 2012). Here, animals received a single acute dose of vehicle or one of the three agents, and samples were collected at time points when organ injury was the most extensive. Serum chemistry and microscopic analysis were used to determine the extent of tissue damage. The time‐matched miRNA expression profile of all samples was then determined using miR‐seq urine, plasma and tissue.

## Materials and methods

### Animals

Eight‐week‐old male Sprague–Dawley rats were purchased from Charles River Laboratories and acclimated to their surroundings for 5 days. The environmental conditions of the animal facility were: a 12: 12 h light/dark cycle; a temperature of 18–29 °C; a relative humidity of 50 ± 20%; and ventilation changes of ≥10 per hour. Animals were supplied with an autoclaved pellet diet (Lab Diet® no. 5002; PMI Nutrition International LLC, St. Louis, MO, USA) and *ad libitum* with reverse osmosis‐filtered water. Animals were randomly assigned to groups by a weight‐ordered distribution before dosing and individually housed in wire‐bottom stainless steel suspended caging. All animal experiments for this study were conducted in accordance with Millennium Pharmaceutical's Institutional Animal Care and Use Committee Guidelines.

### Dosing

Eight rats each received one of the following: (1) 5 ml intravenous administration of 2 or 5 mg kg^−1^ cisplatin or vehicle (0.9% sterile saline); (2) 10 ml oral gavage of 400 or 1250 mg kg^−1^ APAP or vehicle (0.5% methylcellulose); or (3) 10 ml oral gavage of 250 or 1500 mg kg^−1^ CCL_4_ or vehicle (corn oil). Cisplatin was purchased from Teva Pharmaceuticals (Cambridge, MA, USA) while both APAP and CCL_4_ were purchased from Sigma‐Aldrich (St. Louis, MO, USA). Four animals from each group were euthanized 24 h post‐dose and the remaining four animals were euthanized at 72 h post‐dose. Animals first received intramuscular injection of an anesthetic cocktail (75 mg kg^−1^ ketamine HCl, 2.5 mg kg^−1^ xylazine and 2.5 mg kg^−1^ acepromazine) and exsanguinations were performed in accordance with accepted American Veterinary Medical Association guidelines.

### Biological sample collection

The day before scheduled death, rats were transferred to metabolic cages designed for urine collection. Animals were fasted overnight but had access to water. Urine was collected at approximately 24 (*n* = 8) and 72 (*n* = 4) h post‐dose in individual bottles on wet ice that were underneath cages. Urine was centrifuged (1000 *g* for 1 min at 4 °C) to remove debris, aliquoted and stored at ≤ − 70 °C. Urine creatinine was measured with a Beckman Coulter AU680 Chemistry System (Danvers, MA, USA) by Idexx Laboratories (Grafton, MA, USA), and urinary KIM‐1 was quantified with the Kidney Injury Panel 1 (rat) Kit (Meso Scale Diagnostics, Rockville, MD, USA) using a Meso Scale Discovery Sector Imager 2400 following manufacturer's instructions. Raw KIM‐1 values were then normalized to urine creatinine.

Whole blood was collected and processed into both serum and plasma from either the lateral tail vein (non‐terminal) or vena cava (terminal). To obtain serum, blood was collected into tubes containing no anticoagulant, allowed to clot and centrifuged at 4 °C. For plasma, blood was collected into tubes containing ethylenediaminetetraacetic acid and centrifuged at 4 °C. Serum was analyzed with a Beckman Coulter AU680 by Idexx Laboratories for serum chemistry markers of kidney damage (blood urea nitrogen and serum creatinine) or liver damage (alanine transaminase [ALT] and aspartate transaminase [AST]).

Kidney and liver samples were collected at necropsy at 24 and 72 h post‐dose and rinsed in sterile saline. For miRNA analysis, tissues were snap frozen in liquid nitrogen and stored at ≤ − 70 °C. For microscopic evaluation, tissues were fixed in 10% neutral buffered formalin, embedded in paraffin, sectioned at 4–6 μm, mounted on glass slides, stained with hematoxylin and eosin and analyzed by a qualified veterinary pathologist. Microscopic findings were reported in concordance with the standardized nomenclature for classifying lesions within the kidney (Frazier *et al.*, 2012) and liver (Thoolen *et al.*, 2010) of rats.

### RNA isolation and miRNA analysis

Total RNA was isolated from 200 μl plasma or urine by Asuragen (Austin, TX, USA) following a proprietary small‐scale biofluid RNA isolation procedure. Samples were eluted in 20 μl of water to 10 urine equivalents or plasma equivalents per μl. The quality of miRNAs was determined by a proprietary quantitative reverse transcription–polymerase chain reaction (PCR) panel of three miRNAs. Normalization between samples was done using U6 spliceosomal RNA as a housekeeping control. The relative abundance of miRNAs in relation to U6 spliceosomal RNA was calculated using the dCt method (Vandesompele *et al*., 2002).

Isolation of miRNA from 50 μm tissue samples used a lysis buffer (RLT [Qiagen, Hilden, Germany] + 1% β mercaptoethanol) and a rotor‐stator homogenizer. RNA was extracted from tissue homogenate with KingFisher Pure RNA Tissue Kit (Thermo Scientific, Cambridge, MA, USA). Briefly, tissue homogenates were combined with magnetic beads and ethanol, and loaded on to a KingFisher Magnetic Particle Processor (Thermo Scientific). Samples were DNase‐treated, washed and eluted in RNase‐free water. RNA samples were only used in subsequent analysis if they had both a 260: 280 measurement >1.6 and a RNA integrity number > 6.0, as measured by a Caliper LabChip GX (Perkin‐Elmer, Waltham, MA, USA).

Samples from three animals within each treatment were analyzed for changes in miRNA levels using miR‐seq. A TruSeq Small RNA Library Kit (Illumina, San Diego, CA, USA) was used for library construction of rat miRNA. Sequencing was performed on the GAIIx (Illumina) at 36 base pair read length and targeting 12 million reads per sample. Adaptor sequences were clipped and OSA v4 (http://omicsoft.com/osa) was used to align the reads to the rat genome (Rnor_5.0) and to MiRBase (v.21) (Kozomara & Griffiths‐Jones, 2014) allowing for two mismatches and excluding any reads that aligned to >10 genomic locations. Expression levels were quantified by counting the number of reads aligned to mature miRNA region. Differential miRNA analysis was calculated using the DESeq2 generalized linear model (Love *et al.*, 2014). This algorithm was designed for high‐throughput sequencing data that determined fold‐change and statistical significance with a Wald test based on a negative binomial distribution of read counts. Comprehensive data files used for the miR‐seq analysis were uploaded to the Gene Expression Omnibus database (accession number GSE79017). A total of 832 different miRNAs were detected by miR‐seq across all samples.

## Results

### Cisplatin caused kidney damage 72 h post‐dose while both acetaminophen and carbon tetrachloride damaged livers at 24 h post‐dose

**Figure 1:**
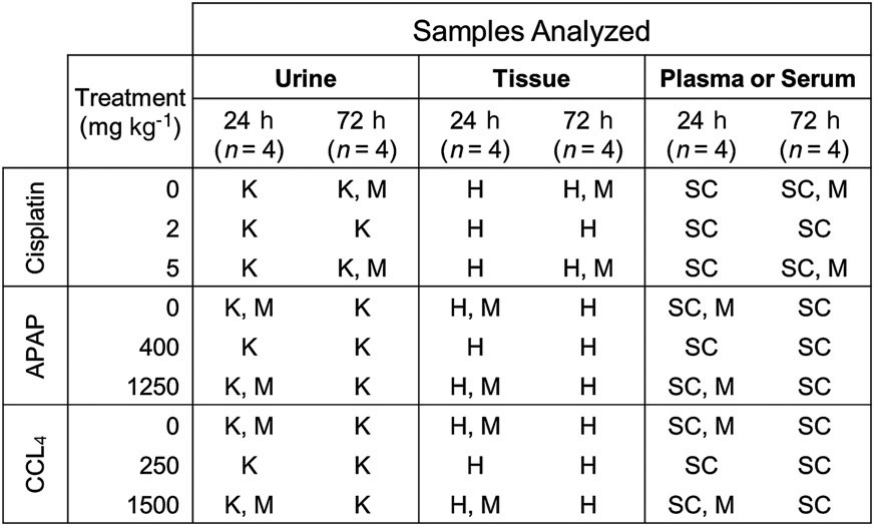
Scheme for sample collection. Urine, tissue, plasma and serum were collected from rats dosed with vehicle or toxicants at the indicated time points. The following assays were performed: K, KIM‐1 urinary analysis; H, histopathology; M, miR‐seq; SC, serum chemistry. APAP, acetaminophen; CCL_4_, carbon tetrachloride.

An analysis of serum chemistry and evaluation of microscopic damage was necessary to understand how the various agents damaged tissues. Briefly, eight Sprague–Dawley rats received a single administration of one of three vehicles, cisplatin, APAP or CCL_4_. Four animals within each group were euthanized at 24 h post‐dose, while the remaining four were euthanized at 72 h post‐dose. A breakdown of the sample collection and analysis scheme is presented in Fig. 1. Traditional serum chemistry and histopathology were initially used to define the severity and specificity of organ injury. Upon completion of this analysis and identification of the dose and time point that corresponded to maximal damage, frozen plasma aliquots were analyzed by miR‐seq.

**Figure 2:**
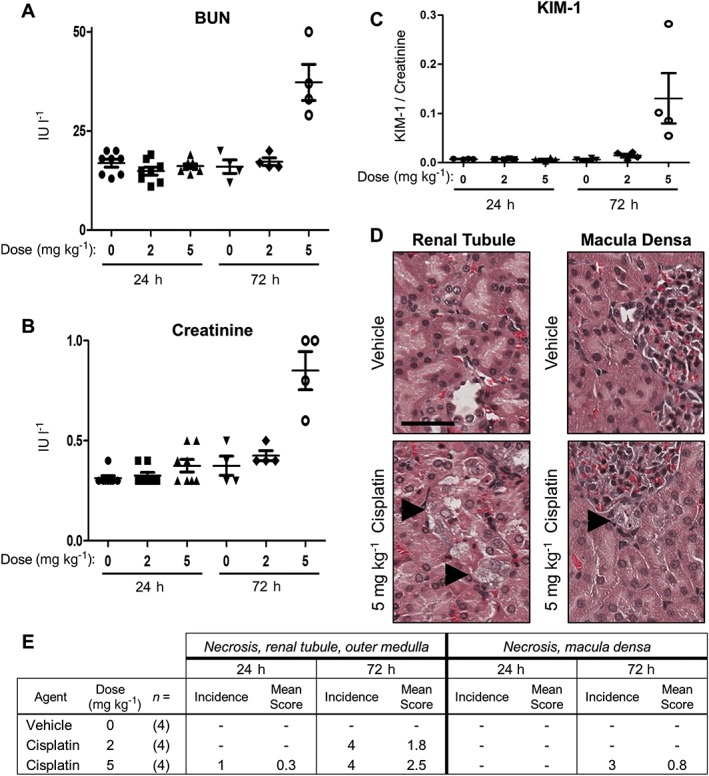
Cisplatin caused maximal kidney damage at 72 h post‐dose. Rats received vehicle, 2 or 5 mg kg^−1^ cisplatin and were assessed for kidney damage at 24 and 72 h post‐dose. (A) Serum BUN. (B) Serum creatinine. (C) Urinary KIM‐1 (ng ml^−1^) was normalized to urinary creatinine. (D) Representative histological images of control and treated kidney renal tubule (left) and macula densa (right) sections. Black arrowheads indicate necrosis, and the black bar is 50 μm. (E) Incidence and severity of kidney microscopic findings. Findings were graded on a scale of 0 (absent), 1 (minimal), 2 (mild), 3 (moderate), or 4 (marked). BUN, blood urea nitrogen.

Animals were dosed with either 2 or 5 mg kg^−1^ cisplatin and the kidney effects were evaluated. These doses were selected based on results from a previous rat miRNA study that used single doses of cisplatin (Kanki *et al*., 2014). The 5 mg kg^−1^ cisplatin dose, but not 2 mg kg^−1^, caused a significant elevation in serum blood urea nitrogen (Fig. 2A) and creatinine (Fig. 2B) that was highest at 72 h post‐dose. Urinary KIM‐1 is a protein marker of kidney damage (Han *et al.*, 2002). Levels of KIM‐1, normalized against urinary creatinine, were highest 72 h after the 5 mg kg^−1^ cisplatin dose (Fig. 2C). Representative histological images of 72 h post‐dose control and cisplatin‐treated kidney tissues illustrate renal tubule necrosis of the outer medulla (Fig. 2D). The incidence and severity of kidney findings are presented in Fig. 2(E). Damage was primarily observed in the 72 h post‐dose cohort. The 5 mg kg^−1^ cisplatin group had the greatest level of necrosis of the outer medulla of the renal tubule and of the macula densa. Vehicles did not cause any damage and neither dose of cisplatin resulted in liver damage (Supplementary Fig.S1.

**Figure 3:**
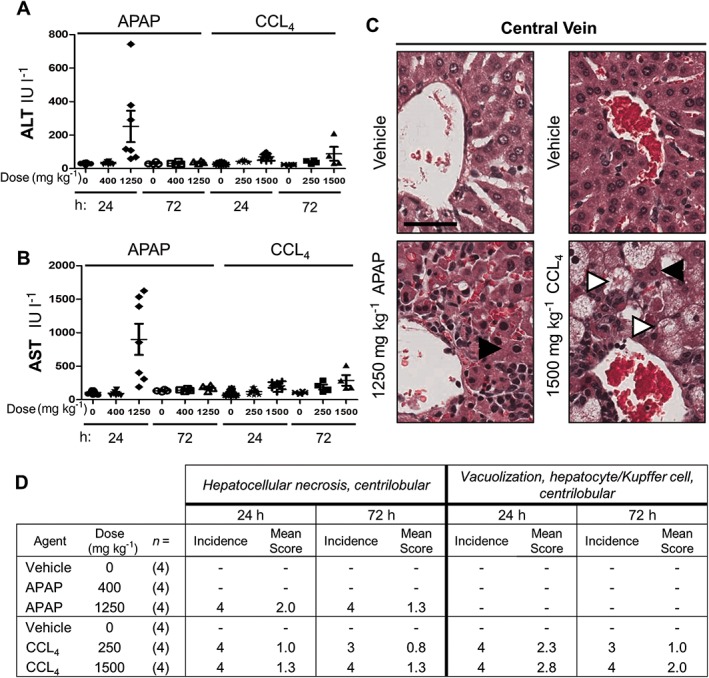
APAP and CCL_4_ caused maximal liver damage at 24 h post‐dose. Rats received the indicated concentrations of vehicle, APAP or CCL_4_ and were assessed for liver damage at 24 and 72 h post‐dose. (A) Serum ALT. (B) Serum AST. (C) Representative histological images of control and treated liver central vein sections. Black arrowheads indicate necrosis, white arrowheads indicate vacuolization; and black bar 50 μm. (D) Incidence and severity of liver microscopic findings. Findings were graded on a scale of 0 (absent), 1 (minimal), 2 (mild), 3 (moderate) or 4 (marked). ALT, alanine aminotransferase; APAP, acetaminophen; AST, aspartate aminotransferase; CCL_4_, carbon tetrachloride.

To induce hepatotoxicity, animals received either 400 or 1250 mg kg^−1^ APAP, or 250 or 1500 mg kg^−1^ CCL_4_. The doses were selected based results from a previous rat miRNA study that used single doses of these compounds (Yang *et al*., 2012). The liver effects were assessed by serum chemistry and histopathology at 24 and 72 h post‐dose. In 1250 mg kg^−1^ APAP‐dosed animals, serum levels of the liver damage markers ALT and AST were respectively greater at 24 h (252 and 902 IU l^−1^) compared to 72 h post‐dose (38 and 175 IU l^−1^) (Fig. 3A,B). Levels of ALT (71 and 89 IU l^−1^) and AST (208 and 288 IU l^−1^) were similar at the 24 and 72 h, respectively, in animals dosed with 1600 mg kg^−1^ CCL_4_ (Fig. 3A,B). Representative histological images of livers from vehicle or either treatment illustrate necrosis (Fig. 3C; black arrowheads) and vacuolization (CCL_4_ only; white arrowheads) proximal to the central vein. These findings indicate that damage caused by both APAP and CCL_4_ was more severe at 24 h than at 72 h, where it appeared to be resolving (Fig. 3D). The lower doses of APAP and CCL_4_ were not further evaluated. Vehicles did not cause any damage and there were no test article‐related kidney findings (Supplementary Fig.S1).

### Treatment with cisplatin, acetaminophen and carbon tetrachloride caused changes in miRNAs from tissue, plasma and urine

The combination of serum chemistry and histopathology demonstrated that the treatments were organ‐specific and established the progression of the injury over time. Samples (kidney, plasma and urine) were analyzed by miR‐seq from 72 h post‐cisplatin animals, and samples (liver, plasma and urine) were analyzed from 24 h post APAP or CCL_4_ animals. The criteria used to determine significant changes were Log_2_ expression fold changes ≥1.5 with *P* < 0.05, which were consistent with criteria used in previous rat miRNA studies (Nassirpour *et al*., 2014; Yang *et al*., 2012). For simplicity, the stem–loop names and fold changes of miRNAs are presented in the main text, and only elevated miRNAs are reported for urine and plasma. Complete details of mature miRNA isoforms, statistical *P* values, gene families and accession numbers are included in Supplementary Table S1.

There were 832 different miRNAs detected by miR‐seq across all samples (Supplementary Table S1). While 455 miRNAs were shared in common with this miR‐seq and the standard TLDA card (TaqMan Array Rodent MicroRNA A + B Cards Set v3.0 [Thermo Scientific]), 377 were detected by miR‐seq but not present on the TLDA card (Supplementary Fig.S2). An additional 181 miRNAs were represented on the TLDA card but not detected by miR‐seq. Given that the objective of this study was to identify novel miRNA biomarkers, miR‐seq appeared to show the greatest potential and was used for all analyses.

Cisplatin caused significant changes in the levels of six miRNAs in the urine, 14 in the plasma and five in the kidney (Table 1). Rats dosed with APAP had significant changes in the levels of six miRNAs in the urine, 74 in plasma and five in liver; for CCL_4_ the changes were six, 20 and five, respectively. Volcano plots of every miRNA detected in each sample are shown for cisplatin (Supplementary Fig.S3), APAP (Supplementary Fig.S4) and CCL_4_ (Supplementary Fig. S5).

**Table 1:** Significantly altered rat miRNAs across treatments and samples

5 mg kg^−1^ Cisplatin				1250 mg kg^−1^ APAP			
Kidney		Plasma		Liver		Plasma	
miRNA	Fold‐change	miRNA	Fold‐change	miRNA	Fold‐change	miRNA	Fold‐change
rno‐miR‐34a	2.36	rno‐miR‐34c	3.14	rno‐miR‐92a	1.67	rno‐miR‐410	5.35
rno‐miR‐146b	1.66	rno‐miR‐128	2.40	rno‐miR‐155	1.66	rno‐miR‐29c	5.23
rno‐miR‐34c	1.53	rno‐miR‐34a	2.09	rno‐miR‐21	1.65	rno‐miR‐582	5.08
rno‐miR‐144	−1.53	rno‐miR‐130b	2.01	rno‐miR‐223	1.62	rno‐miR‐30a	4.92
rno‐miR‐451	−1.94	rno‐miR‐702	1.82	rno‐miR‐146b	1.56	rno‐miR‐125b	4.89
		rno‐miR‐6215	1.75			rno‐miR‐107	4.47
Urine		rno‐miR‐484	1.74	Urine		rno‐miR‐466c	4.32
		rno‐miR‐134	1.69			rno‐miR‐505	3.80
rno‐miR‐378a	6.12	rno‐let‐7e	1.66	rno‐miR‐320	12.05	rno‐miR‐466b	3.68
rno‐miR‐1839	3.82	rno‐miR‐151	1.63	rno‐miR‐126a	5.96	rno‐miR‐3559	3.62
rno‐miR‐140	3.39	rno‐miR‐191a	1.59	rno‐miR‐185	5.75	rno‐miR‐221	3.53
rno‐miR‐26b	3.07	rno‐miR‐431	1.55	rno‐miR‐130b	4.93	rno‐miR‐1249	3.52
rno‐let‐7 g	2.40	rno‐miR‐181b	1.52	rno‐miR‐148a	4.07	rno‐miR‐499	3.22
rno‐miR‐22	1.77	rno‐miR‐92b	1.51	rno‐miR‐3473	2.22	rno‐miR‐31a	3.19
						rno‐miR‐674	3.17
				Plasma		rno‐miR‐455	3.17
						rno‐miR‐101a	3.12
				rno‐miR‐802	175.75	rno‐miR‐195	3.08
				rno‐miR‐122	112.42	rno‐miR‐21	3.07
				rno‐miR‐320	65.50	rno‐miR‐671	3.04
1500 mg kg^−1^ CCL_4_				rno‐miR‐192	61.41	rno‐miR‐29a	3.00
				rno‐miR‐193a	45.58	rno‐miR‐26b	2.76
Liver		Plasma		rno‐miR‐194	29.51	rno‐miR‐374	2.75
				rno‐miR‐200a	26.49	rno‐miR‐497	2.70
miRNA	Fold‐change	miRNA	Fold‐change	rno‐miR‐182	25.36	rno‐miR‐30c	2.68
				rno‐miR‐22	17.89	rno‐miR‐34a	2.64
rno‐miR‐146b	4.18	rno‐miR‐122	27.09	rno‐miR‐365	17.73	rno‐miR‐27b	2.63
rno‐miR‐223	2.59	rno‐miR‐802	15.99	rno‐miR‐200b	17.25	rno‐miR‐30d	2.62
rno‐miR‐155	2.30	rno‐miR‐193a	5.88	rno‐miR‐429	15.96	rno‐miR‐30e	2.61
rno‐miR‐342	1.50	rno‐miR‐192	4.49	rno‐miR‐183	15.29	rno‐miR‐361	2.42
rno‐miR‐1247	−1.61	rno‐miR‐194	4.30	rno‐miR‐378b	14.66	rno‐miR‐1843a	2.41
		rno‐miR‐182	4.13	rno‐miR‐101b	13.00	rno‐miR‐155	2.37
Urine		rno‐miR‐365	3.52	rno‐miR‐378a	12.04	rno‐miR‐664	2.30
		rno‐miR‐200b	3.23	rno‐miR‐99a	11.47	rno‐let‐7 g	2.27
rno‐miR‐450a	10.22	rno‐miR‐22	3.03	rno‐miR‐362	9.33	rno‐miR‐144	2.26
rno‐miR‐92a	5.08	rno‐miR‐183	2.86	rno‐miR‐592	8.18	rno‐miR‐148a	2.23
rno‐miR‐184	3.89	rno‐miR‐101b	2.76	rno‐miR‐375	7.62	rno‐miR‐103	2.10
rno‐miR‐3473	2.75	rno‐miR‐200a	2.76	rno‐miR‐152	7.42	rno‐miR‐1839	2.09
rno‐let‐7i	2.67	rno‐miR‐31a	2.47	rno‐miR‐598	7.09	rno‐miR‐210	1.98
rno‐miR‐10b	2.04	rno‐miR‐429	2.34	rno‐miR‐193b	6.60	rno‐let‐7f	1.92
		rno‐miR‐155	2.29	rno‐miR‐1247	6.47	rno‐miR‐345	1.91
		rno‐miR‐19a	1.91	rno‐miR‐665	6.34	rno‐miR‐872	1.82
		rno‐miR‐20a	1.82	rno‐miR‐23b	6.20	rno‐let‐7a	1.71
		rno‐miR‐21	1.72	rno‐miR‐293	6.00	rno‐miR‐20a	1.70
		rno‐miR‐532	1.57	rno‐miR‐203b	5.72	rno‐let‐7c	1.62
		rno‐miR‐340	1.56	rno‐miR‐141	5.48		

APAP, acetaminophen; CCL_4_, carbon tetrachloride.

The stem–loop names of significantly altered miRNAs (Log_2_ expression fold changes ≥1.5 with *P* < 0.05) from tissue, urine and plasma are as shown.

### Cisplatin treatment caused a sixfold elevation of urinary miR‐378a

**Figure 4:**
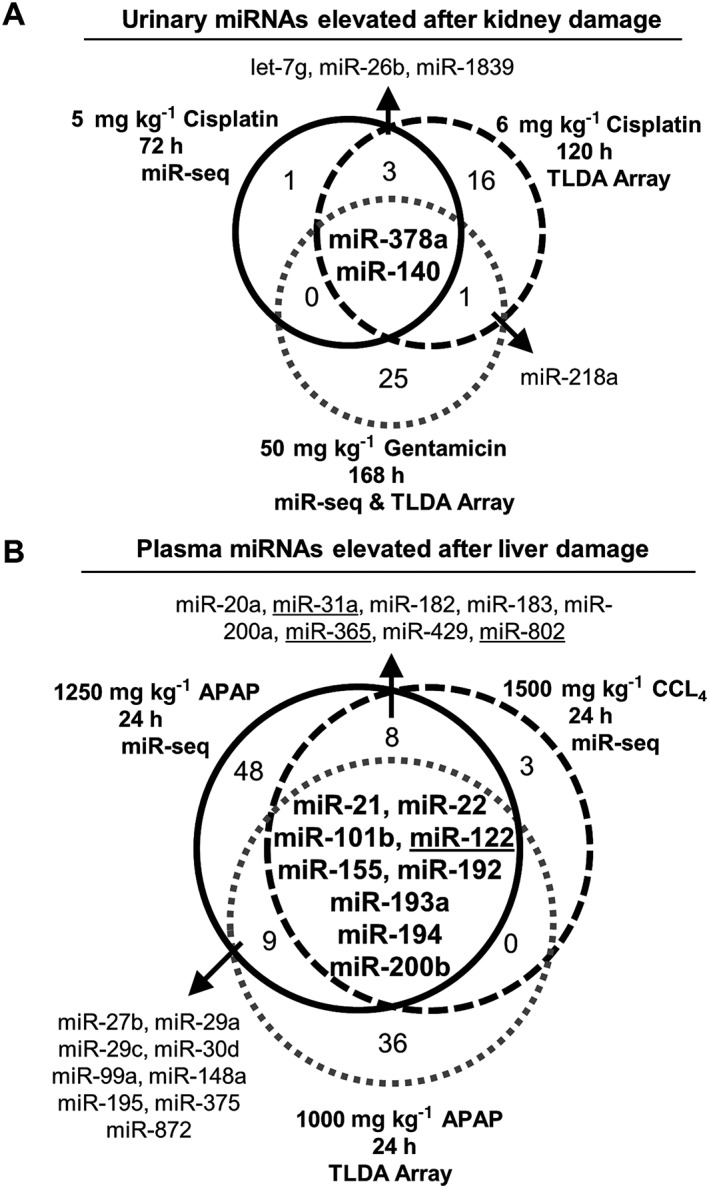
Elevated miRNAs in urine after kidney damage and in plasma after liver damage. Venn diagrams illustrate overlapping miRNAs from this study with other published data. Only the stem–loop miRNA identifiers were used due to differing annotation methods across studies. Dose of toxicant, time point sampled and technological platform (miR‐seq and/or a TLDA card) are indicated. Numbers within each circle represent different miRNAs. (A) Elevated miRNAs observed in this study after cisplatin treatment (black circle) and two other studies by Kanki *et al.* (2014; black dashed circle) and Nassirpour *et al*. (2014 *.* (B) Elevated miRNAs observed in this study after a single dose with APAP (black circle), CCL_4_ (black dashed circle), or in an APAP study by Yamaura *et al.* (2014. Four underlined miRNAs were identified by the Smith *et al.* (2016) rat miRNA atlas as being liver specific/enriched. APAP, acetaminophen; CCL_4_, carbon tetrachloride; TLDA, TaqMan low‐density rodent miRNA array.

There were six elevated miRNAs in the urine after cisplatin treatment. Of these, five were also elevated in a second rat model (Kanki *et al*., 2014) of cisplatin toxicity (Fig. 4A). When compared to a third study using the nephrotoxicant gentamicin (Nassirpour *et al*., 2014), only miR‐378a (up 6.1‐fold) and miR‐140 (up 3.4‐fold) were in common in all studies. None of the 11 different miRNAs elevated in the urine after APAP/CCL_4_ (Table 1) treatment had reported hepatic origins or were enriched in the rat liver (Smith *et al*., 2016). A single mature miRNA, miR‐320‐5p, was elevated in both the plasma and urine of rats dosed with APAP, but little is known about the function/specificity of this miRNA other than it is expressed in rat gastrointestinal tract (Smith *et al*., 2016).

### Treatment with acetaminophen and carbon tetrachloride caused an increase in liver‐specific miRNAs in the plasma

Numerous miRNAs were detected in the plasma after treatment with the three toxicants (Table 1). There were 17 elevated miRNAs in common between the APAP/CCL_4_ treatments (Fig. 4B), some of which overlapped with another rat APAP model (Yamaura *et al.*, 2014). Whenever possible, miRNAs between studies were aligned by mature isoforms, but if that information was lacking, the stem–loop name was used to make comparisons. These 17 miRNAs correspond to 23 different mature miRNA isoforms (Supplementary Fig. S6). Of these, miR‐122‐5p, miR‐122‐3p, miR‐31a‐5p, miR‐365‐3p, miR‐802‐5p and miR‐802‐3p (Fig. 4B, underlined) were specific to the rat liver (Smith *et al*., 2016). Of note, miR‐122‐3p and miR‐802‐3p are not included on the TaqMan Array Rodent MicroRNA A + B Cards Set v3.0. Previous versions of this technology do not differentiate well between the stem–loop and mature miRNA names. For cisplatin, none of the 14 elevated miRNAs were enriched in the rat kidney (Smith *et al*., 2016).

### Altered miRNAs in kidney or liver tissue after treatment

Kidney or liver injury resulted in few significantly altered miRNAs (Table 1). Expression of miR‐146b was elevated in kidney/liver tissues 1.6–4.2‐fold after each treatment. This miRNA has reported roles in hematopoiesis (Zhai *et al.*, 2014) and in liver fibrosis (Ge *et al.*, 2014) but is not specific to the rat liver (Smith *et al*., 2016). Within the kidney, while the 2.4‐fold increase of miR‐34a was consistent with previous other rodent models of cisplatin toxicity (Bhatt *et al.*, 2010; Pavkovic *et al.*, 2014), this miRNA is also expressed in many other tissues (Misso *et al.*, 2014; Smith *et al*., 2016). Both APAP and CCL_4_ treatments resulted in a 1.6–2.6‐fold elevation of miR‐155 and miR‐223 in the liver. However, others have suggested that these two miRNAs may be linked to lymphocyte infiltration into areas of damage, so their origin is unclear (Yamaura *et al*., 2014). None of the remaining miRNAs are known to be specific to the kidney or liver (Smith *et al*., 2016).

## Discussion

Rodents are routinely used in non‐clinical investigations to determine the toxicological effects drugs have on the body. Efforts to improve on the traditional serum chemistry markers used to evaluate organ damage have focused on miRNAs. The work described here evaluated miRNA biomarkers of acute kidney and liver damage in Sprague–Dawley rats. Plasma, and not serum, was analyzed to keep findings consistent and comparable to other published studies (Church *et al*., 2016; Yamaura *et al.*, 2012, 2014). Significantly elevated plasma and urine miRNAs in identified in this study were compared to published rat studies and to the recently completed rat and dog miRNA atlases. We concluded that miR‐378a is a novel urinary biomarker of kidney damage, while miR‐122, miR‐31a, miR‐365 and miR‐802 are plasma markers of liver injury.

One challenge in identifying novel miRNA biomarkers of injury is that many studies report conflicting data. Our twofold approach to provide more confidence in identifying novel biomarkers was to (1) compare our data for one type of organ injury against other studies, and (2) cross‐reference the rat miRNA atlas (Smith *et al*., 2016). For example, in the study presented here, APAP/CCL_4_ treatment caused elevation of both the miR‐155 and miR‐223 in the plasma. This finding is of questionable significance given that these two miRNAs have been erroneously identified as “specific” across numerous published studies (Haider *et al.*, 2014). A second example comes from a study that measured urinary miRNAs in rats with liver damage (Yang *et al*., 2012). Only miR‐182 overlapped between that study and the work presented here, but this miRNA is not enriched in the rat liver (Smith *et al*., 2016). It is also unclear how miRNAs from tissues not connected to the renal system could enter the urine, as the kidney may serve as a barrier between blood and urinary miRNAs (Weber *et al.*, 2010). There remains considerable doubt that urinary miRNAs have any utility as biomarkers for liver damage.

miR‐378a was primarily detected in skeletal muscle of rats and to a lesser extent the kidneys (Smith *et al*., 2016). However, this miRNA was one of the highest expressed miRNAs in the entire dog kidney (Koenig *et al*., 2016). In humans, miR‐378a has also been used as a serum biomarker of renal disease (Hauser *et al.*, 2012; Redova *et al.*, 2012; Wang *et al.*, 2015). Here, cisplatin caused an elevation of miR‐378a in the urine of rats (Table 1), which was also seen in two other studies (Kanki *et al*., 2014; Nassirpour *et al*., 2014). All three studies identified kidney tubule damage through the use of histopathology. Taken together, miR‐378a may have utility as a marker of renal tubule damage. Follow‐up investigations are critical to determine whether there is translatability from animals to humans. Of note, miR‐140 was also elevated in the urine of multiple rat nephrotoxicity studies, but was not enriched in either rat or dog kidneys, and thus needs further characterization to evaluate its utility as a biomarker.

The plasma miRNAs with the three highest fold changes in this study were miR‐122, miR‐802 and miR‐192, which respectively represent an established, a putative and a poor biomarker of liver damage. First, miR‐122 is a known biomarker of hepatotoxicity in rats (Smith *et al*., 2016; Su *et al.*, 2012; Yamaura *et al.*, 2012), dogs (Harrill *et al*., 2014; Koenig *et al*., 2016) and humans (Wang *et al*., 2009). In this study miR‐122 was elevated a respective 112‐ and 27‐fold after treatment with APAP or CCL_4_ compared to controls (Table 1). Second, miR‐802 was elevated 176‐ and 16‐fold after APAP or CCL_4_ treatments, and in rats was recently shown to be enriched in the liver (Smith *et al*., 2016) and was elevated after hepatobiliary injury (Church *et al*., 2016). Third, while miR‐192 was elevated 4–61‐fold in this study, it is highly expressed across multiple tissue types, such as the kidney (Mladinov *et al.*, 2013) and the rat gastrointestinal tract (Smith *et al*., 2016). Despite being reported otherwise, miR‐192 may not be a strong candidate biomarker due to its limited tissue specificity. Subsequent validation work is necessary to determine which organ‐specific miRNAs can be used as part of a panel approach to assess tissue injury.

It is unclear why the expression levels of so few miRNAs were significantly altered in tissues, particularly considering that dozens of miRNAs were elevated in the plasma after treatment. The most likely explanation is that this study assessed miRNA changes across whole organs, not just the damaged portions. An improvement to the study design would involve microdissection of areas of damage that could be isolated for subsequent miR‐seq analysis. As a proof of concept, different miRNA expression patterns were identified within the renal cortex and medulla of the dog kidney (Ichii *et al.*, 2014). A second explanation for the scarcity of altered tissue miRNAs is that the time points used for kidney and liver damage were not optimal in capturing the profiles of miRNAs involved in the necrosis and regeneration biological processes. Taken together, none of the significantly altered tissue miRNAs seems to have utility as specific biomarkers of injury at the time points examined in this study.

There are considerable challenges when comparing data from one rat biomarker study to another. At the broadest level, the strain, gender and age of rats influence their basal miRNA expression. Once collected, a urine sample can be normalized measuring creatinine (Nassirpour *et al*., 2015) or volume (Kanki *et al.*, 2014). Plasma is typically normalized against a housekeeping miRNA (Church *et al*., 2016; Yang *et al*., 2012). There are additional hurdles related to the detection technology, such as older generations of TLDA cards that use outdated miRNA nomenclature that make it hard to know whether “miR‐802” refers to the −5 or the −3 mature isoform. A similar problem was demonstrated in one study that used only the “A” card to assess urinary miRNA biomarkers and not the “B″ card that contains the assay for miR‐378a (Nassirpour *et al.*, 2015). For miR‐seq, there are questions about how to normalize data. We used the highly cited DESeq2 generalized linear model (Love *et al*., 2014), which was also used in an earlier rat miR‐seq biomarker study (Nassirpour *et al.*, 2014). Taken together, while miR‐seq has a great ability to detect novel miRNA biomarkers, there certainly remains value in using TLDA arrays or quantitative PCR to validate results.

One key lesson learned from these studies regards the importance of selecting appropriate time points for sample collection. For example, liver damage caused by APAP was rapid and began to resolve at the end of the study while the CCL_4_ damage was less severe but lingered. An interesting follow‐up study would be to assess differences in miRNA expression between acute and chronic exposures to these toxicants. Assessing miRNA changes at multiple time points might have provided information about both the initial injury and the healing process. An added benefit of this approach would be to create a stronger model for chronic human renal and hepatic diseases. The next critical step is to evaluate these biomarkers in additional non‐clinical studies with different toxicants to determine how well they can be used to evaluate organ damage.

## Conflict of interest

The authors did not report any conflict of interest.

## Supporting information

Supporting info item

Supporting info item

Supporting info item

Supporting info item

Supporting info item

Supporting info item

Supporting info item

Supporting info item
